# Validation of an LC-MS/MS Method for the Determination of Abscisic Acid Concentration in a Real-World Setting

**DOI:** 10.3390/foods12051077

**Published:** 2023-03-03

**Authors:** Elisabetta Schiano, Ilaria Neri, Maria Maisto, Ettore Novellino, Fortuna Iannuzzo, Vincenzo Piccolo, Vincenzo Summa, Lucia Grumetto, Gian Carlo Tenore

**Affiliations:** 1 Department of Pharmacy, University of Naples Federico II, via Domenico Montesano 59, 80131 Naples, Italy; 2 Department of Medicine and Surgery, Catholic University of the Sacred Heart, 00168 Rome, Italy

**Keywords:** abscisic acid, waste-fruit products, glucose homeostasis, method validation, liquid chromatography, mass spectrometry

## Abstract

One of the most relevant aspects in evaluating the impact of natural bioactive compounds on human health is the assessment of their bioavailability. In this regard, abscisic acid (ABA) has attracted particular interest as a plant-derived molecule mainly involved in the regulation of plant physiology. Remarkably, ABA was also found in mammals as an endogenous hormone involved in the upstream control of glucose homeostasis, as evidenced by its increase after glucose load. The present work focused on the development and validation of a method for the determination of ABA in biological samples through liquid–liquid extraction (LLE), followed by liquid mass spectrometry (LC-MS) of the extract. To test method suitability, this optimized and validated method was applied to a pilot study on eight healthy volunteers’ serum levels to evaluate ABA concentration after consumption of a standardized test meal (STM) and the administration of an ABA-rich nutraceutical product. The results obtained could meet the demands of clinical laboratories to determine the response to a glucose-containing meal in terms of ABA concentration. Interestingly, the detection of this endogenous hormone in such a real-world setting could represent a useful tool to investigate the occurrence of impaired ABA release in dysglycemic individuals and to monitor its eventual improvement in response to chronic nutraceutical supplementation.

## 1. Introduction

2-cis, 4-trans-abscisic acid (ABA) is a sesquiterpenoid phytohormone synthesized via an indirect pathway from the cleavage products of carotenoids [1]. This molecule has been studied for several decades with regard to its pivotal role as a regulator of plant growth and response to abiotic and biotic stress [2,3]. Due to ABA involvement as a growth regulator, immature fruits have been found to contain the highest concentration of this phytohormone [4,5] in the context of vegetal matrices. In this regard, a screening of various immature fruits derived from fruit thinning has identified thinned nectarines (TN) as the richest source of this bioactive compound [6]. Nevertheless, ABA has sparked particular interest not only as a phytohormone commonly found in vegetables and fruits, but it has also been found in mammals as an endogenous hormone involved in the upstream control of glucose homeostasis [7,8,9] via interaction with its specific receptor lanthionine synthase C-like 2 (LANCL2) [10].

To date, the majority of evidence for the hypoglycemic effects of ABA in vivo has addressed a role in the stimulation of peripheral glucose uptake by increasing the expression and translocation of glucose transporter 4 (GLUT4) [11,12,13,14]. In addition, it is noteworthy to remark that in patients with type 2 diabetes mellitus (T2DM) or gestational diabetes, a decreased release of ABA have been found following a glucose load [15]. This evidence further strengthens the importance of monitoring serum concentrations of ABA in individuals with altered glucose metabolism and supplementing them with plant-based exogenous sources of ABA. In this context, several studies involving both animal and human models demonstrated the significant beneficial effects of ABA-containing nutraceuticals on the glycemic profile in prediabetic and diabetic subjects, in association with an insulin-sparing mechanism of action [6,14,16,17,18,19]. In virtue of its insulin-independent mechanism of action [13], ABA supplementation may be indicated as a useful approach to improve glucose tolerance in individuals with insulin deficiency in and/or insulin resistance. In this regard, there is a growing scientific consensus that sustained stimulation of insulin release from pancreatic β-cells under conditions of chronic hyperglycemia may ultimately contribute to their depletion [20]. In view of this evidence, hypoglycemic molecules able to decrease glycemia without increasing insulinemia are highly desirable as they could improve the survival and function of pancreatic β-cells. On the other hand, although a wide variety of bioactive compounds of natural origin have been tested for their beneficial potential in the control of diabetic conditions [21,22], the evaluation of their bioavailability still represents a crucial aspect [23,24]. Identification of ABA as a plant hormone is usually performed with various methods, mainly in plant matrices, such as gas chromatography/mass spectrometry (GC/MS) [25] and immunological assay, i.e., enzyme-linked immunosorbent assay (ELISA) [26]. Although these methods are able to assess ABA concentration levels, they are affected by some disadvantages. For instance, ELISA assay requires a long preparation time and has low specificity and reproducibility, while GC/MS requires derivatization of the sample [25]. Based on such considerations, the present work focused on the development and validation of a method for the determination of ABA by liquid mass spectrometry analysis (LC-MS), through liquid–liquid extraction (LLE) in a biological matrix, i.e., serum. Subsequently, the optimized and validated method was applied to test its suitability on serum samples from eight healthy volunteers that consumed a standardized test meal (STM) with the concomitant supplementation with a nutraceutical product based on TN rich in ABA, to test the method in a real-world setting. Finally, the glycemic and insulinemic response in the above-mentioned subjects was evaluated in association with ABA serum levels at different time points of analysis.

## 2. Materials and Methods

### 2.1. Study Design

#### 2.1.1. Participants and Standardized Test Meal Composition

Briefly, healthy subjects of both sexes were recruited in May 2019 by Samnium Medical Cooperative (Sant’Agata De’ Goti, Italy) as a subset of volunteers participating in a randomized clinical trial. The volunteers’ letter of intent, the protocol, and the synoptic documents of the study were submitted to the Scientific Ethics Committee of AO Rummo Hospital (Benevento, Italy). The study was approved by the committee (protocol no. 28, 15 May 2017) and was conducted in accordance with the Helsinki Declaration of 1964 (as revised in 2000). The study was listed on the ISRCTN registry (www.isrctn.com, accessed on 24 June 2022) with ID ISRCTN16732651. A total of 8 healthy subjects aged 18–83 years were invited to participate. Exclusion criteria were diabetes mellitus (DM) type 1 and type 2, liver, heart, or renal disease, drug therapy or intake of dietary supplements containing ABA, underweight (body mass index < 18.5 kg/m^2^), pregnancy or suspected pregnancy, birch pollen allergy. All participants received oral and written information about the study before giving written informed consent. Before inclusion in the study, volunteers were subjected to self-reporting questionnaires involving the following items: residence, occupation, smoking status, alcohol consumption, drug administration, and dietary habits. The volunteers meeting the inclusion criteria (body mass index 27–35 kg/m^2^; waist circumference, men ≥ 102 cm and women ≥ 88 cm) were assigned to consume a standardized test meal (STM), immediately after the administration of TN (1 g, lyophilized) containing 15 µg of ABA, as reported in our previous work [6]. TN treatment was self-administered as a tablet. The STM composition consisted of white bread (100 g) with 50 g of jam and 100 g of mozzarella and 200 mL of fruit juice. These amounts were chosen based on indications of a balanced meal, as they provided 50% of calories from carbohydrates, 20% from protein, and 30% from fat [27].

#### 2.1.2. Experimental Procedures

At the beginning of the study, the height, body weight, and waist circumference (WC) of all patients were measured and the Body Mass Index (BMI) was determined. Glucometabolic parameters were determined before the STM consumption as baseline, except for fasting plasma glucose (FPG) and fasting plasma insulin (FPI), which were evaluated before and after consuming the STM. After a 12-h fasting period, blood samples were collected to measure FPG, FPI, triglycerides (TG), total plasma cholesterol (TC), lipoprotein-cholesterol (HDL-C), alanine aminotransferase (ALT), aspartate aminotransferase (AST), and glycated hemoglobin (HbA1c). The concentration of the above-mentioned parameters was assayed by enzymatic colorimetric methods (Diacron International, Grosseto, Italy). The Friedewald formula was used to calculate LDL cholesterol levels. Plasma insulin levels were measured by ELISA (DIA-source ImmunoAssay S.A., Nivelles, Belgium) on a Triturus analyzer (Diagnostic Grifols S.A., Barcelona, Spain). HbA1c was measured using a commercially available kit (InterMedical s.r.l, Grassobbio, Italy).

### 2.2. Analytical Method

#### 2.2.1. Chemicals and Reagents

The purity of ABA as primary standard was ≥98% HPLC and purchased from Sigma-Aldrich (Milan, Italy). Chromatographic-grade solvents, methanol, formic acid, and ethyl acetate were used (minimum purity 99.9%) and purchased by Sigma Aldrich, (Milan, Italy) as well as internal standard (IS), bis 4,4′- Sulfonyldiphenol, (BPS), (minimum purity 98%). Ultra-purified water Milli Q was produced in-house (conductivity 0.055 μS cm^−1^ at 25 °C, resistivity equals 18.2 MΩ·cm). 

#### 2.2.2. Real Sample Preparation and Extraction

Vacu-test^®^ tubes were employed to collect blood samples, collected from the antecubital vein (5 mL); the samples were immediately centrifuged at 2200 rpm for 20 min and the supernatant was frozen and stored at −80 °C until processing. Both samples, synthetic and real samples, underwent liquid–liquid extraction (LLE). Briefly, the sample preparation was performed according to the following procedures: 75 µL of serum were transferred to a 2 mL vial, spiked into 40 µL of BPS 100 ppb solution, to achieve a final concentration of 40 ppb, with the addition of 340 µL of methanol and 2 µL of 12 N HCl solution. Each sample was successively vortexed and stored in ice for 2 min. Afterwards, the samples were added to 500 µL ethyl acetate, vortexed, and finally centrifuged at 10.000 rpm for 5 min at 4 °C. The supernatant (a fixed volume of 700 µL) was transferred to a 4 mL vial, dried in Savant™ SpeedVac™ (Thermo Scientific™, Hyannis, MA, USA) and stored until the analysis. Dried samples were dissolved in 50 μL of CH_3_OH:H_2_O 50/50 *v*/*v*, vortexed, and after 45 min to facilitate the dissolution, another 50 μL of CH_3_OH:H_2_O 50/50 *v*/*v* was added. The samples were again centrifuged at 3.500 rpm for 5 min and the supernatant was transferred to a 1.5 mL glass insert and injected into liquid mass spectrometry (LC-MS). BPS was chosen for its lipophilicity feature as an internal standard (IS) to assess the recovery of each extraction. 

#### 2.2.3. Equipment

Analytical determination was performed on an Ultimate 3000 LC system (Dionex/Thermo Scientific™, San Jose, CA, USA) coupled to a linear ion trap LTQ XL™ (Thermo Scientific™, San Jose, CA, USA), with an electrospray ionization source. The separation was performed on Luna^®^ Omega 3 µm Polar C18 column (100 × 2.1 mm) (Phenomenex Torrance USA, Torrance, CA, USA). Tuning and data acquisition were carried out using Xcalibur and quantification using Qual Broswer software 4.4 version.

#### 2.2.4. LC-MS/MS Conditions

The samples, 5 μL of each, were injected from the Autosampler (Ultimate 300) and analyzed under the following chromatographic conditions: eluent A aqueous added to 0.1% *v*/*v* formic acid and eluent B acetonitrile, added to 0.1% *v*/*v* formic acid, flow rate set to 0.4 mL min^−1^, at a room temperature of 35 ± 2 °C. Gradient elution was accomplished as follows: 0–2.0 min, 5% B; 2.0–9.0 min, 95% B; 9.0–12.0 min, 95% B; 12.1–16.0  min, 5% B. All mobile phases were vacuum-filtered through 0.45 μm nylon membranes (Millipore^®®^, Burlington, MA, USA). The electrospray ionization (ESI) mass spectrometer (MS) was operated in negative ion mode using selective reaction monitoring (SRM) with nitrogen as the nebulizer, auxiliary, collision, and curtain gas. The main working source/gas parameters of mass spectrometer were optimized and maintained as follows: curtain gas, 8; nebulizer gas, 8. The instrumental parameters employed were as follows: ESI spray voltage in the negative-ion mode, 4 kV; sheath gas flow-rate, 70 arb; auxiliary gas flow-rate, 20 arb; capillary voltage, −38 V; capillary temperature, 350 °C; and tube lens, 95 V. ABA was monitored as [M-H]^−^ ion according to its *m*/*z* values.

#### 2.2.5. Calibration Curve and Linearity

European validation guidelines were followed to validate the method [28]. Stock solutions of ABA were obtained dissolving the reference standard in 100% methanol to obtain a final concentration of 2.000 ppm. Five solutions with different concentrations (40 ppb, 20 ppb, 10 ppb, 4 ppb, 2 ppb) were prepared by diluting this stock. The linearity ranges were tested using the average peak areas against the concentration (ppm) of ABA. Linear regression analysis and calibration curve parameters (Coefficient of Determination R^2^, slope, and intercept) were back-calculated from the peak areas using the regression line by the method of least squares, and mean accuracy values were determined [29,30].

#### 2.2.6. Limits of Detection (LOD) and Quantification (LOQ)

LOD and LOQ were estimated as the concentrations providing signals equal to 3 and 10 times, respectively. They were calculated based on the following equations: LOD = SD∙3/S and LOQ = SD∙10/S [31], where SD is the standard deviation of the intercept response with the y-axis of the calibration curves, and S is the slope of a calibration curve. The spike level was 2 ppb in the appropriate range using a concentration and was assessed by running the measurement ten times.

#### 2.2.7. Precision and Accuracy

The method’s precision was evaluated by running five replicates of the sample repeated in the same day and in two different days to cover both intra-day and inter-day precision, expressed as relative standard deviation (RSD%). Repeatability was assessed using the nominal concentration of ABA (2 ppb). The accuracy of this method was determined considering samples spiked with 2–40 ppb of ABA (quality control samples, QCs) and evaluated at each level in triplicate, and reported as a percentage of the nominal value. 

#### 2.2.8. Selectivity

Serum working calibration standards were prepared using sera already present in the archive of our laboratory and processed for other research, to assess the absence of ABA and that any signal interfered with the retention time of ABA. These sera, considered as blanks, were also employed to optimize the extraction process. 

#### 2.2.9. Carry-Over

Carry-over effect of the method was evaluated by injecting methanol solvent after running the highest concentrated samples of ABA spiked in the serum (three times) and observing the occurrence of signals within the retention windows of the target chemicals.

#### 2.2.10. Matrix Effect

The matrix effect was investigated by calculating the ratio of the peak area in the presence of the matrix (matrix spiked with ABA post extraction) to the peak area in the absence of the matrix (ABA in methanol) [32]. The serum matrix blank was spiked with the analyte at each concentration of the linear range (2 ppb, 4 ppb, 10 ppb, 20 ppb, and 40 ppb). The ratio was calculated as follows:

(1) $$\mathrm{Matrix}\;\mathrm{effect}\;\%=\frac{\mathrm{peak}\;\mathrm{area}\;\mathrm{in}\;\mathrm{presence}\;\mathrm{of}\;\mathrm{matrix}}{\mathrm{area}\;\mathrm{in}\;\mathrm{absence}\;\mathrm{of}\;\mathrm{matrix}}\cdot 100$$

#### 2.2.11. Recovery

The recovery was assessed by evaluating the relative abundance of the BPS peak (I.S.) spiked before the extraction procedure and calculated as follows:

(2) $$\mathrm{Recovery}\;\%=\frac{\mathrm{found}\;\mathrm{concentration}}{\mathrm{standard}\;\mathrm{concentration}}\cdot 100$$

The results of the real samples were corrected for the recovery.

## 3. Results

### 3.1. Anthropometric and Glucometabolic Parameters

The characteristics of the patient population at baseline are shown in Table 1. A total ofeight healthy adults (three men and five women) aged 18 to 45 years with a BMI between 18 and 25 kg/m^2^ met the inclusion criteria and were therefore eligible to participate in the study. The group was well balanced in terms of demographic and clinical factors.

### 3.2. Two-Hour Glycemic and Insulinemic Responses to Standardized Test Meal

**Figure 1:**
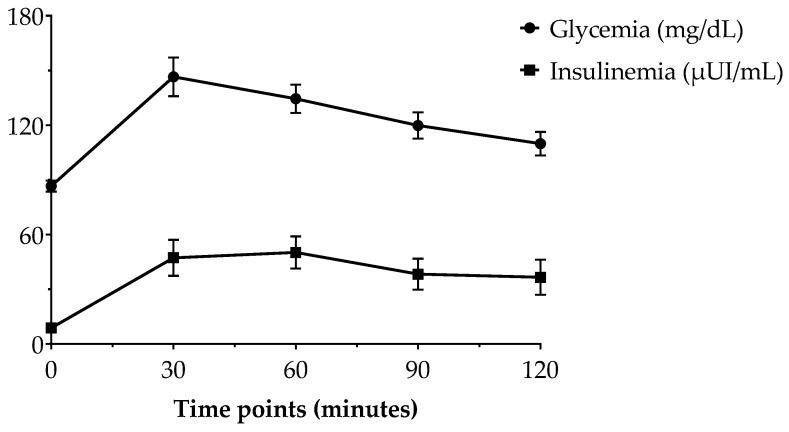
Change in postprandial plasma glucose and insulin concentration in healthy adults (n = 8). Data are expressed as mean ± SEM.

**Figure 2:**
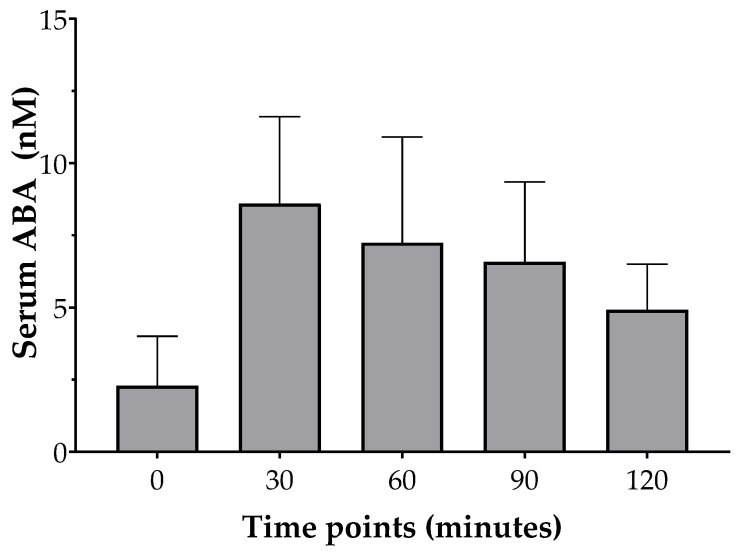
ABA concentration change after the consumption of STM and ABA-rich nutraceutical product in healthy adults (n = 8). Data are expressed as mean ± SEM.

Following the STM, which was preceded by administration of the nutraceutical supplement containing ABA, mean plasma glucose levels reached a peak at 30 min and gradually decreased to pre-prandial levels by 120 min. According to the plasma glucose response curve, the post-prandial insulinemic response curve peaked at 30 min and gradually declined to the pre-prandial level by 120 min (Figure 1). A similar trend can be observed for serum ABA concentrations after the consumption of STM and ABA-rich nutraceutical product in volunteers under our investigation, as shown in Figure 2.

### 3.3. Optimization of Chromatographic Method

Different “synthetic” samples with known ABA concentrations, i.e., methanolic solutions and serums spiked with ABA, were used for the method development. These samples were subjected to the above-mentioned method in order to evaluate the efficiency in isolating and detecting abscisic acid in the context of complex biological matrices. The proposed method of extraction and quantification of ABA was easy to handle and sensitive to the analysis in serum matrix, optimizing the method after several changes in operating. For the extraction procedures, there were distinct organic solvents in various percentages with water. Ethyl acetate as an extraction solvent was the most efficient solvent to extract ABA from the serum matrix (data not shown). The spike levels (40.0 ppb and 2.0 ppb) were in the recommended range, i.e., calculated LOD < spike level < 10 × calculated LOD. For LC-MS analysis, we optimized the method using different stationary reversed-phases (Luna^®®^ Omega 3 µm Polar C18 column (100 × 2.1 mm) (Phemomenex Torrance USA) and an Inertsil ODS-3 column (2.1 mm × 100 mm, 5 μm) (Torrance, CA, USA), and by a varying gradient elution program, to achieve an adequate resolution for the two analytes from the interferents. Optimal transitions were obtained for ABA (C_15_H_20_O_4_, MW: 264.32 g/mol) at *m*/*z* 152.000, and for BPS (C_12_H_10_O_4_S, MW: 250.27 g/mol) at *m*/*z* 107.000. The linear R-squared values (r^2^ = 0.9981) show a good linearity in the range of the calibration curves performed in the serum matrix from 40 ppb to 2 ppb. The sensitivity of the developed method is appreciable from the listed LOD and LOQ parameters, with values of 1.59 ppb and 5.31 ppb, respectively. The RSD% of within-run precision was 2.30%, while the RSD% between-run precision was 12.01%. Repeatability was performed using the repeatedly frozen and thawed ABA samples, and we did not observe any differences in the raw data and degradation products. Recovery from the serum matrix, evaluated at high and low spiking concentrations (40 ppb and 2.0 ppb), resulted in 70.3%. Matrix effect was 39.97% and variations in the experimental parameters did not result in any appreciable change in the method performance. Table 2 summarizes all method validation parameters. 

**Figure 3:**
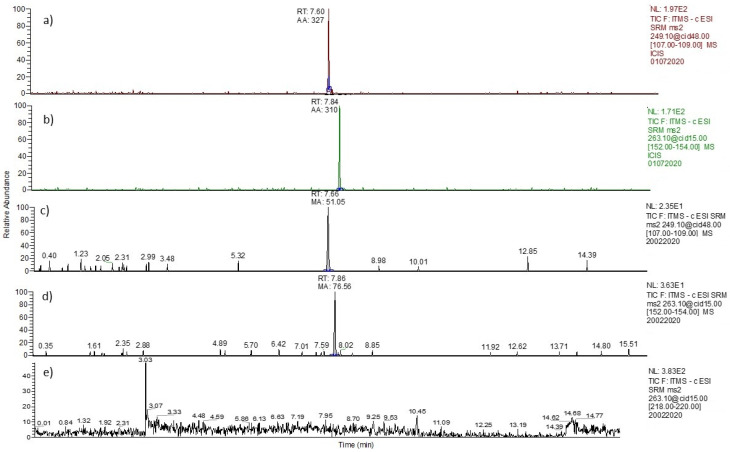
Chromatogram of (**a**) BPS in solvent, (**b**) ABA in solvent, (**c**) spiked BPS in serum (2 ppb), (**d**) spiked ABA in serum (2 ppb), and (**e**) blank serum sample.

These results demonstrate that the analytical method developed provides a reliable response relevant to the analysis of ABA in such a complex biological matrix. Selectivity is the ability of an analytical method to differentiate and quantify the analyte in the presence of other components in the sample. The selectivity of the method was evaluated by analyzing a blank sample, compared to a blank sample spiked with the lower limit of quantification LOQ (ABA equal to 2.00 ppb). As can be seen in Figure 3, the selectivity of this method was good.

## 4. Discussion

In the present work, a method for the determination of ABA in biological samples by liquid–liquid extraction (LLE), followed by liquid mass spectrometry (LC-MS) of the extract, was developed and validated. The above-reported method has significant advantages, as it does not require expensive operations, in terms of procedures and amounts of solvents used, and leads to results with a good level of accuracy, reproducibility, LOD and LOQ values. These results are better in terms of sensitivity than those achieved by Reverse-Phase HPLC-DAD analysis on food and beverage matrices [33]. Moreover, to the best of our knowledge, the scientific literature reports methods for determination of ABA by LC/MS, but in a matrix other than serum, such as in *Arabidopsis thaliana* [31], Rose Leaf Samples [32], and fresh Oryza sativa tissues [33]. The scientific works analyzing ABA in the serum matrix are not focused on the validation method, and therefore, do not report validation parameters for comparison [34,35]. Anyway, new strategies to detect ABA with high sensitivity are under development, as fluorescent probes, but performed on plant tissues [36].

Moreover, the application of the optimized method on serum samples of healthy volunteers who consumed a STM together with a nutraceutical product rich in ABA allowed us to evaluate its applicability in a suitable biological model. Accordingly, the STM composition of the present work provided 50% of calories from carbohydrates, 20% from protein, and 30% from fat, in agreement with the guidelines for balanced nutrition [27]. In this manner, the glycemic and insulinemic response, together with the increase in plasmatic ABA, was evaluated in the closest to real-life setting. 

The LC-MS analysis performed on the serums obtained from the eight volunteers showed different ABA levels at each time point. As observed in Figure 2, the found data confirmed the involvement of this endogenous hormone in the human response to glucose-containing foods. For all subjects, indeed, the serum ABA levels reached the highest concentration 30 min after the consumption of the STM and the nutraceutical product based on TN. In this regard, various studies carried out on human serums attempted to identify and quantify ABA levels, by performing different isolation and detection methods [11,15]. Specifically, plasma ABA levels have been shown to increase in normal glucose tolerant (NGT) subjects following an oral glucose load [14], but not in patients with T2D or in pregnant women with gestational diabetes mellitus (GDM). On the other hand, resolution of GDM one month after childbirth is associated with a restoration of the ABA response to glucose load [15]. Interestingly, a significant increase in ABA was observed in obese patients after biliopancreatic diversion (BPD), a bariatric surgery performed to reduce body weight and improve glucose tolerance, compared to pre-surgery levels [15]. 

Another observed difference between T2D and NGT individuals was related to fasting ABA values, which were significantly higher in T2D compared to NGT subjects (1.15 vs. 0.66 as median values, respectively). Nevertheless, the distribution of ABA values was found to be normal in NGT but not in T2D patients [15]. These alterations could be due to the heterogeneity of ABA-related dysfunction that occurs in T2D, such as the inability of ABA to increase in response to hyperglycemia or resistance to the activity of ABA. Overall, these observations suggest a role for ABA as a key hormone involved in the management of glucose homeostasis and highlight the importance of monitoring ABA levels in these groups of individuals. Notably, based on reports about daily consumption of fruits and vegetables containing ABA, epidemiological evidence indicates that the majority of the population assumes a very low intake of ABA from dietary sources [37]. Due to the multiple positive health effects attributed to the role of ABA [38], interest in supplementing this bioactive molecule through the administration of nutraceutical products rich in ABA is increasing over time, also in view of the nanomolar blood concentrations of this hormone required for its efficacy.

## 5. Conclusions

In conclusion, we herein developed and validated a method for the extraction and LC-MS/MS analysis of ABA in biological samples. Even if limited by the small sample size, requiring therefore confirmation through larger clinical evaluation, an added value is represented by the successful application of this method to real samples, which allowed the evaluation of ABA serum changes after the consumption of STM and an ABA-rich nutraceutical product. Overall, the results shown could provide a starting point for determining the response to a glucose-containing meal in clinical practice, in terms of ABA concentration. Indeed, serum detection of this endogenous hormone may be considered as a marker to assess the presence of an impaired ABA response in dysglycemic subjects. Undoubtedly, the use of this analysis would be of great interest for clinical trials involving the chronic administration of ABA-rich nutraceutical supplements with hypoglycemic potential.

## Figures and Tables

**Table 1:** Baseline anthropometric and glucometabolic parameters of study participants.

Characteristics	Study Participants (n = 8)
**Demographic and anthropometric parameters**	
Male sex (No (%))	3 (37.5%)
White ethnicity (No (%))	8 (100%)
Age (years)	34 ± 3.7
Height (m)	1.6 ± 0.9
Weight (kg)	71.2 ± 6.9
BMI (kg/m^2^)	24.6 ± 3.3
WC (cm)	89.4 ± 5.4
**Clinical parameters**	
HbA_1c_ (%)	6.5 ± 0.4
Triglycerides (mg/dL)	105.2 ± 14.5
Total cholesterol (mg/dL)	171.3 ± 11.9
HDL-C (mg/dL)	46.7 ± 5.4
LDL-C (mg/Dl)	89.5 ± 7.6
AST (UI/L)	25.6 ± 3.9
ALT (UI/L)	18.7 ± 2.8
Creatinine (mg/dL)	0.9 ± 0.1

Data are expressed as mean ± standard deviation. Abbreviations: AST: aspartate aminotransferase; ALT: alanine aminotransferase; BMI: Body Mass Index; Cre: creatinine; F: females; FPG: fasting plasma glucose; FPI: fasting plasma insulin; HbA1c: glycated hemoglobin; HDL-C: high density lipoprotein-cholesterol; LDL-C: low density lipoprotein-cholesterol; M: males; TG: triglycerides; WC: waist circumference.

**Table 2:** Summary of method validation parameters. Recovery was reported for each concentration of the linear range, and also reported as mean %.

Linear Range (ppb)	Slope	Intercept	R2	Repeatability (n = 5) RSD %	Intermediate Precision (n = 10) RSD%	LOQ (ppb)	LOD (ppb)	Matrix Effect
40.0–2.0	92.84	−32.45	0.9981	2.30	12.01%	5.31	1.59	39.97%
Spiking level (ppb)	2	4	10	20	40			
Recovery (%)	71.8	73.3	77.7	65.5	63.5			

## Data Availability

The data used to support the findings of this study are included within the article.
